# Feline obesity is associated with stronger owner attachment, while indoor confinement increases risk of obesity at an early age in domestic shorthaired cats

**DOI:** 10.3389/fvets.2026.1757719

**Published:** 2026-03-18

**Authors:** Charlotte Reinhard Bjørnvad, Camilla Brohave Mortensen, Maria Louise Støvlbæk Tams, Freja Kragh Jørgensen, Peter Sandøe, Thomas Bøker Lund

**Affiliations:** 1 Department of Veterinary Clinical Sciences, University of Copenhagen, Frederiksberg, Denmark; 2 Department of Food and Resource Economics, University of Copenhagen, Frederiksberg, Denmark; 3 Department of Veterinary and Animal Sciences, University of Copenhagen, Frederiksberg, Denmark

**Keywords:** body condition score, overweight, outdoor access, feeding management, Lexington Attachment to pets scale

## Abstract

Knowledge of risk factors for overweight and obesity is important for making preventative strategies for feline obesity. The present study investigated risk factors for feline obesity in privately owned adult cats on Zealand, Denmark. Privately owned cats (>1 year old and reportedly healthy) were recruited through social media. During home visits, the cats underwent a full physical examination and body condition was scored by trained investigators. Owners answered a questionnaire relating to cat characteristics, owner characteristics and attachment to their cat, feeding practices and living environment. For statistical analyses cats were divided into moderately lean to moderately overweight (BCS 4–6/9) and heavy/obese (BCS 7–9/9) groups. Multivariable logistic analysis was performed to predict risk of a cat being heavy/obese. 192 cats were included in the final analysis. 65% were BCS 4–6/9, and 35% were BCS 7–9/9. In the multivariate analysis, owners of heavy/obese cats were significantly more attached to their cat and owners of these cats perceived their cat as less active than owners of normal weight cats. Indoor confined domestic shorthaired cats had a high risk of being heavy/obese from a young age (1 year old), while the risk for domestic shorthaired cats with outdoor access was low at a young age and only slowly increased – culminating around 7 years of age. In contrast, in purebred cats, age only modestly affected the risk of being heavy/obese. In conclusion, indoor confinement was identified to significantly increase the predicted risk of domestic shorthaired cats being heavy/obese from a young age, while the risk for cats having outdoor access was highest at an age around 7 years. Whether a closer owner attachment is a contributing factor in feline obesity development should be investigated further.

## 1 Introduction

Excessive weight is a major health concern in companion cats (*Felis catus*), associated with an increased risk of several debilitating diseases (1–4). Across the globe there have been reports of high levels of overweight and obesity with a reported prevalence of up to 63% (2–12). Weight loss is achievable in companion cats, but the success rate of feline weight loss programs is limited, often followed by a subsequent weight regain (13–15). Therefore, effective preventative strategies are needed. A first step in developing these strategies is to identify specific risk factors and life stages affecting energy requirement and intake. Based on this, it will be possible to develop risk factor based feeding guidelines for use in private practice that can support efforts to prevent development of feline overweight and obesity.

Several risk factors of feline obesity have been identified in previous studies, and though there is not full agreement on all, some risk factors seem to be well established. Identified cat related risk factors include male sex, neutering, young to middle-age and domestic shorthair (DSH) or mixed breed (2, 3, 8, 9, 16–20). Furthermore, indoor housing/apartment dwelling without outdoor access is a frequently reported risk factor (6, 16, 21, 22). Feeding related risk factors are less clear and differ between studies. Some studies identified feeding dry food (16, 21, 23, 24), therapeutic or light diet (19) and canned food *ad libitum* (22) while other studies have not found a relation with diet type (2, 5, 8, 12, 25, 26). Feeding treats or table scraps has been identified as a risk factor in some studies (22, 24, 27) but not in others (8, 12, 23, 25, 26). One study found feeding according to the cats appetite and begging behavior a risk factor (23) while another study found highly indulgent feeding to be protective (16). Owner related risk factors are less studied, but one study identified playing with the cat as a protective factor (28) while a recent international survey found no association with owner attachment (16) using the Lexington Attachment to pets scale (LAPS) (29). Finally, one study identified having an owner that found chubby cats to be cute as a risk factor (23).

Most studies, investigating body composition among cat populations are designed as questionnaire studies based on convenience sampling, typically via social media, and they rely on owner reported evaluation of their cats’ body composition. Sampling of owners via social media prompts for various biases, notably a selection bias towards very committed cat owners; and owner assessed body-composition is inherently unreliable, with several studies finding, that owners will report their cat to be normal weight when veterinary professionals evaluate them to be either under- or over-weight (9, 25, 30). Other studies are based on cats recruited at veterinary practices. This recruitment strategy enables a more uniform and accurate body condition scoring, but unfortunately, it may induce a different selection bias, because many cats are infrequently brought to the veterinarian unless they show signs of disease (31).

One study from 1992 evaluated body composition in Danish cats presenting at a single private practice (18). Here, the reported prevalence was 28% for overweight and 13% for obesity, and neutering and indoor confinement were identified as risk factors. A more recent representative questionnaire-based study, focusing on the welfare of cats in Denmark, found an owner reported overweight and obesity prevalence of 10.5% (31). It is likely that owners have underestimated the BCS of their cats, but the sample differed from other studies of companion cats, as the majority (69%) of the cats in the representative population were classified as having free outdoor access and only 31% were indoor housed or had limited outdoor access, and this could be a contributing factor to the low prevalence of overweight.

In the present cross-sectional study, we wished to investigate the prevalence and risk factors for feline obesity in privately owned adult cats examined by expert assessors in their home environment in Denmark. The following risk factors were examined: (1) cat characteristics (2) owner characteristics and attachment to the cat, and (3) feeding and housing management. Furthermore, we studied (4) whether interactions between cat characteristics, owners’ attachment status and feeding and management regimes served to explain the cats’ weight status. Compared to previous studies the strength of the current study is the objective assessment of the weight status of the cats.

## 2 Materials and methods

### 2.1 Participants and recruitment

The study was approved by the local administrative and ethical committee at the Department of Veterinary Clinical Sciences, Faculty of Health and Medical Sciences, University of Copenhagen (approval number 2018-4), and data collection took place during the period 1st March to 31st May 2018. Cats were included if they were adult (>1 year of age), reportedly healthy and receiving no medication. Exclusion criteria were gestation and known chronic disease that could affect body condition, such as inflammatory bowel disease, chronic kidney disease, diabetes mellitus and hyperthyroidism.

The study was cross-sectional and based on a convenience sample of cats owned by owners responding to an advertisement through the University hospital Facebook feed, investigators’ private feed and designated Danish cat interest group feeds. To achieve variation in terms of the socio-economic profile of the cat owners as well as the living environment of the cats, cats living in the capital region of Copenhagen as well as suburban and rural areas on Zealand where recruited. If there were more than one cat in the household, only the cat closest to 5 years of age was included. Owners of eligible cats were asked to sign an informed written consent if they agreed to participate in the study and home visits were arranged. During home visits, two final year veterinary master students (CBM and MLST) performed a full physical examination including weighing on a calibrated baby scale and BCS scoring, and owners filled out the questionnaire.

### 2.2 Procedures

Body condition scoring was performed according to the 9-point BCS system validated for cats (32) adapted to the World Small Animal Veterinary Association’s (WSAVA) toolkit (33), where a score is given based on palpation and visual inspection of the cat’s ribs, waist, bony prominences, tail base and abdominal tug. As this system is subjective and to some degree operator-dependent, it is important to calibrate the scoring procedure across investigators. Prior to recruitment, the two student investigators (CBM and MLST) were therefore trained in body condition scoring by a veterinary nurse and a veterinarian (CRB) both experienced in the procedure. The BCS training was undertaken at a cat shelter (Kattens Værn, Brøndby, Denmark). The training was performed on 19 cats of varying ages and a BCS range of 4–8/9, mainly DSH cats.

During the home visit, owners completed a questionnaire produced in the software program SurveyXact (Rambøll Management Consulting, Copenhagen, Denmark). The questionnaire (Supplementary Table S1) included information relating to cat characteristics (age, sex, neuter status, age at neutering and breed), owner characteristics such as socio-demographic information (for example level of education, gender, current work situation and household income), the owners’ height and weight as well as cat management factors (such as outdoor access, cat activity level, number of cats in household and feeding practices). Finally, the owners’ emotional attachment to their cat was evaluated using the Lexington Attachment to pets scale (LAPS) (29). Following completion of the questionnaire, the cat underwent a full physical examination, was weighed and the student investigators (CBM/MLST) individually assigned a BCS of 1–9/9 (33). In case of disagreement between the students, the cat was reevaluated, findings discussed and a mutual agreement was reached.

### 2.3 Measures

An overview of the measures along with descriptive statistics is provided in Tables 1, 2. The measures included in the statistical analyses are described in more detail below.

**Table 1:** Body condition score (BCS, proportions and average) and obesity status in a sample of privately owned cats (*n* = 199).

Body condition score	*N*	%
1	0	0
2	0	0
3	6	3
4	22	11.1
5	45	22.6
6	58	29.1
7	33	16.6
8	20	10.1
9	15	7.5
Average BCS (standard deviation)	6.01	(1.48)
Obese cat (BCS 7–9)	68	34.2

**Table 2:** Descriptive statistics regarding cat and owner characteristics, and cats’ activity levels based on a sample of owners and their cats visited in their home environment across Zealand, Denmark (*N* = 192) age of cat and owner, is presented as median [Range], cats body weight, LAPS and owner assessed cat activity are presented as mean (SD).

Variable	BCS 4–6/9 (*N* = 125)		BCS 7–9/9 (*N* = 67)		*p*-value
	*N*	%	*N*	%	
Cat characteristics					
Sex and neutering status					
Male intact	1	1%	1	2%	0.60
Male neutered	75	60%	37	55%	
Female intact	4	3%	2	3%	
Female neutered	45	36%	27	40%	
Age at neutering (*N* = 120 & 64)					
3–6 months	36	30%	20	31%	0.98
6–9 months	31	26%	16	25%	
9–12 months	19	16%	9	14%	
>12 months	25	21%	14	22%	
Unknown	9	7%	5	8%	
Age (in years)	4.0 [1–15]		4.5 [1–13]		0.91
Breed					
Domestic shorthaired (DSH)	66	53%	47	70%	<0.001
Purebred	37	30%	5	8%	
Mixed breed or unknown	22	17%	15	22%	
Body weight (in kg)	4.8 (1.2)		5.9 (1.1)		<0.001
Owner characteristics					
Gender					
Male	10	8%	12	18%	0.04
Female	115	92%	55	82%	
Age (in years)	41 [16–81]		39 [18–71]		0.37
Education					
Compulsory school (9th—10th grade)	13	10%	4	6%	0.62
High school	6	5%	4	6%	
Vocational	27	22%	18	27%	
Tertiary (2–4 years)	58	46%	28	42%	
Higher tertiary (>4 years)	21	17%	13	19%	
Household income					
0–53,549 Euro	49	39%	25	37%	0.69
53,550–80,319 Euro	20	16%	13	19%	
80,320–120,479 Euro	27	22%	14	21%	
≥120,480 Euro	10	8%	7	11%	
Income unknown	19	15%	8	12%	
Single or two-adult household					
Single	40	32%	19	28%	0.77
Two adults	75	60%	44	66%	
With parents or others	10	8%	4	6%	
Children in household					
No	77	62%	46	69%	0.33
Yes	48	38%	21	31%	
Housing					
Farm/house in countryside	15	12%	10	15%	0.01
House/apartment with garden	76	61%	22	33%	
Apartment/house without garden	30	24%	35	52%	
Other	4	3%	–	–	
Owner weight status					
Normal weight	50	40%	27	40%	0.78
Overweight	44	35%	23	34%	
Obese	19	15%	12	18%	
Not reported	12	10%	5	8%	
Owner attachment to cat (LAPS)^a^	42.8 (13.46)		46.84 (13.21)		0.05
Cat’s activity level (scale 1–10)	6.6 (2.2)		5.3 (1.8)		<0.01
Cat housing					
Indoor or walked on leash	43	34%	39	58%	0.001
Outdoor access	82	66%	28	42%	
Number of cats in the household					
1	67	54%	30	45%	0.07
2	44	35%	23	34%	
3 or more	14	11%	14	21%	

Sample size is 192 unless otherwise stated.

a Lexington Attachment to pets scale (score range: 0–69).

#### 2.3.1 Outcome variable

The measured BCS was the first of two outcome variables used in the analysis. The second outcome variable, overweight status, grouped the raw BCS scores into two categories, indicating whether the cat was normal weight (moderately thin to moderately overweight, BCS 4–6/9) or heavy/obese (BCS = 7–9/9). Cats with BCS < 4/9 were excluded from the statistical analysis as this represents underweight and could indicate subclinical disease. The reason for including cats with a BCS 4/9 and 6/9 in the normal weight group is based on recent studies indicating no or only slight health effects compared with BCS 5/9 or even a protective effect of mild overweight condition on feline longevity (34, 35).

#### 2.3.2 Cat characteristics

The cat characteristics that were taken into consideration in the analysis were the sex, neuter status, breed (DSH vs. purebred), age at neutering (3–6 months, 6–9 months, 9–12 or >12 months of age) and age of the cat.

#### 2.3.3 Owner characteristics and attachment to the cat

A number of socio-demographic characteristics of the owners, including the gender, age and educational qualifications, were included in the analysis. Furthermore, owner weight status was measured through voluntary owner self-reporting of body weight (kg) and height (m). After calculating BMI (BMI = kg/m^2^), the weight status of the owner was categorized into slim/normal-weight (BMI ≤ 25), overweight (25 < BMI < 30), obese (BMI ≥ 30). The LAPS scale was used to assess owner attachment to the cat. This measure has been used for several studies since it was first validated (29). It is a score that is based on 23 questions where owners are asked to grade their level of agreement (strongly agree, somewhat agree, somewhat disagree and strongly disagree) in relation to their own perceived attachment to their cat, their cats’ role in people-substitution and the importance of animals rights/animal welfare (Supplementary Table S1). The scale defines “attachment” in terms of the human emotional tie toward a member of another species, the greater the tie the greater the attachment. Following the guidelines in Johnson et al. (29), including reverse coding of two items, all 23 LAPS items were summated to indicate a single measure of attachment.

#### 2.3.4 The cat’s activity and feeding management

Two measures that may serve to indicate the cat’s activity level were used. The first was Indoor confinement vs. Outdoor access, where the indoor confinement is defined as the cat being kept entirely indoors, only walked on a leash or having outdoor access for short periods (e.g., during vacations), and outdoor access meant that the cat could get access to outdoor roaming either by owner letting them out or having access to a cat flap. Further, owners were asked to rate their cats’ activity level on a scale from 1 to 10 with 1 being inactive and 10 being very active. These responses were transformed to three gradient levels of activity (low: 1–4; medium: 5–7; high: 8–10).

Four measures of feeding management were also used. The first was feeding preference, feeding primarily dry food (>75% of the food) or a combination of dry and wet food. The second was number of daily meals: once a day, twice a day or three times a day or more, or ad libitum, food is always available. The last two measures focused on treats given to the cat, the first whether treats were given daily or less often and finally whether treats were given as add on to the daily ration or integrated in the daily ration of feed.

### 2.4 Data analyses

This study focused on variables at the individual (i.e., the cat and owner) level. Descriptive statistics such as Mean (standard deviation) and frequencies were reported and bivariate tests of associations were calculated using *T*-test and Chi^2^ tests in Excel (Windows 365, Microsoft, USA).

To examine what predicts that a cat is heavy/obese we carried out a multivariable logistic regression [dependent variable: Not heavy/obese = 0 (BCS 4–6); Heavy/obese = 1 (BCS: 7–9)] where the following variables relevant to the cats biological and environmental conditions were included as main effects: the cat’s age, breed, sex, neuter status/timing of neutering, indoor confinement versus outdoor access, the cat’s activity level, number of cats in the household, and feeding management (2 variables: number of daily meals, and treats given to the cat). Further, to examine whether there was a curvilinear association between the cat’s age and risk of being heavy/obese, the squared term of age was included in the regressions. A two-way interaction effect between the cat’s age and indoor confinement versus outdoor access, and an interaction between the squared term of the cats’ age and indoor confinement versus outdoor access were included in the regression. The following owner-related variables were entered: LAPS, gender, education, age, and owner weight status.

Results from the regression analysis are reported using predicted probabilities calculated from Stata’s *margins* command (Stata 17 for Windows, StataCorp LLC, Texas, USA).

## 3 Results

### 3.1 Recruitment, sample composition, body condition and cat characteristics

A total of 200 cats were examined during the study period. One owner failed to complete the questionnaire resulting in a final sample of 199 cats (Table 1). The average BCS in the sample was 6.05 ± 1.47/9 (Table 1). No cats were severely underweight (BCS 1–2/9), while 34% of the cats were heavy/obese (BCS 7–9/9, Table 1). Six cats that were evaluated to be underweight (BCS 3/9) were excluded from the data analyses (Tables 2, 3). One cat (BCS 8/9) was reported to be 20 years old. In comparison to the rest of the cohort, that were all between 1 and 15 years old, the 20-year-old cat was considered an outlier and therefore excluded from the statistical analyses, resulting in a cohort of 192 cats (125 normal weight and 67 heavy/obese) for the final analyses.

**Table 3:** Descriptive statistics regarding feeding practices and owners awareness of overweight from owners and their cats visited in their home environment across Zealand, Denmark (*N* = 192).

Variable	BCS 4–6/9 (*N* = 125)		BCS 7–9/9 (*N* = 67)		*p*-value
	*N*	%	*N*	%	
Number of daily meals					
Once per day	5	4%	6	9%	<0.05
Twice per day	23	18%	21	31%	
Three or more times per day	11	9%	10	15%	
*Ad libitum*	86	69%	30	45%	
Type of food fed to the cat					
Regular/standard food	102	82%	51	76%	0.02
Weight loss diet	4	3%	8	12%	
Prescription diet	19	15%	8	12%	
Feeding ≥75% dry food					
Yes	90	72%	50	75%	0.70
No	35	28%	17	25%	
Have you asked for/received advice on feeding					
No	70	56%	35	52%	0.50
Yes—from veterinarian	38	30%	20	30%	
Yes—from pet shop	5	4%	4	6%	
Yes—from friends and relatives	14	11%	6	9%	
Yes—from internet/books	13	10%	8	12%	
Yes—from diet packaging	6	5%	4	6%	
Yes—other	2	2%	2	3%	
How is diet allocation determined					
According to appetite/begging	37	30%	16	24%	0.51
According to recommendations	20	16%	7	10%	
According to weight change	22	18%	17	25%	
Have never thought about it	27	22%	20	30%	
Other	19	15%	7	10%	
Does your cat receive treats or snacks					
No	38	31%	13	19%	0.50
Yes—daily	39	31%	24	36%	
Yes—weekly	35	28%	18	27%	
Yes—seldom	13	10%	11	16%	
Which of the following fits best?					
Treats are in addition to diet	69	79%	40	75%	0.91
Daily diet allocation is reduced	8	9%	4	8%	
Part of the cats diet is used as treats	3	4%	2	4%	
None of the above	7	8%	7	13%	
Have you discussed your cats weight with the veterinarian					
No	79	63%	38	57%	0.56
Yes—underweight	4	3%	1	2%	
Yes—normal weight	36	30%	7	10%	
Yes—overweight	5	3%	18	27%	
Do not know	1	1%	3	4%	
Did the discussion result in feeding change (*N* = 46 and 29)					
No	35	76%	12	41%	0.004
Yes	11	24%	16	55%	
Do not know	–		1	4%	
Do you consider overweight a problem for cats					
Yes—to a very high degree	19	15%	9	13%	0.05
Yes—to a high degree	48	38%	15	22%	
Yes—to some degree	37	30%	26	39%	
Yes—to a low degree	9	7%	6	9%	
No	1	1%	1	2%	
Do not know	10	7%	10	15%	
If your cat was obese would you adjust the diet allocation					
No	15	6%	9	6%	0.98
Yes	101	90%	51	85%	
Do not know	5	4%	7	9%	
Change to a weight loss diet					
No	15	12%	9	13%	0.71
Yes	101	81%	51	76%	
Do not know	9	7%	7	11%	
Increase your cats activity					
No	28	22%	11	16%	0.23
Yes	81	65%	51	76%	
Do not know	16	13%	5	8%	

The majority of cats were neutered and there was a slight overrepresentation of male neutered cats in both the normal weight and heavy/obese group (Table 2). Approximately half of the neutered cats were neutered before the age of 9 months and there was no difference in age at neutering between the normal weight and heavy groups. As nearly all of the cats were neutered, a statistical evaluation of effect of neutering status was not possible (Table 2). There was no difference between groups for sex and age (Table 2). The majority of cats were between 3 and 11 years old with only two cats being >15 years (Table 2). The purebred cats represented 13 different breeds, with Norwegian Forrest cat, Main Coon and Birman representing more than half of the group. DSH cats, which made up a little more than half of the cohort, were more likely to be heavy/obese compared with purebred cats and the mean body weight of cats in the heavy/obese group was significantly higher than for the normal weight group (*p* < 0.001, Table 2).

### 3.2 Owner characteristics and attachment to cat

Female owners accounted for the majority of the respondents, as seen in other similar surveys (23, 28), but the proportion of male owners were higher in the heavy/obese group (*p* < 0.05, Table 2). There was a considerable span in the educational level of the owners and in household income levels, suggesting that the sample encompassed a wide socio-economic range. Two-adult households were the most common and there were children in approximately one-third of the households. Significantly more owners of cats in the heavy/obese group lived in an apartment/house without garden access compared with the normal weight group (*p* < 0.01, Table 2).

Owners of heavy/obese cats were more attached to their cats based on the LAPS (*p* < 0.05, Table 2). More owners of cats in the normal weight group considered overweight to be a problem for cats to a high or very high degree than owners of heavy/obese cats (*p* < 0.05, Table 3). However, across groups, the majority considered overweight to be a problem to at least some degree and thought that it increased the risk for developing diseases like diabetes mellitus, cardiac disease, joint disease, urinary and liver disease. Most owners responded that they would be willing to decrease their cats diet allocation, change to a weight loss diet and increase their cats’ activity if their cat was obese, and there was no difference between groups (Table 3).

### 3.3 Housing and feeding management

A higher proportion of heavy/obese cats were indoor confined or only walked on a leash, while more normal weight cats had outdoor access, either by being let out by their owner or given access through a cat flap (*p* < 0.001, Table 2). Cats in the heavy/obese group were perceived as less active by their owners compared with cats in the normal weight group (*p* < 0.01, Table 2). The majority of cats in the current cohort were fed *ad libitum,* but significantly fewer cats in the heavy group were fed *ad libitum* compared with normal weight cats (*p* < 0.05, Table 3). For both groups, cats were mainly fed dry food (>75% of the daily diet allocation) and they were primarily fed a regular/standard diet for adult cats (Table 3). A purpose made weight loss diet was seldomly fed to the cats, but it was fed significantly more to cats in the heavy/obese group compared with the normal weight (*p* < 0.02, Table 3). Half of the cat owners had sought advice regarding feeding of their cat. When the owners were asked about where they had sought or received advice on feeding their cat, they were allowed to name more than one source and the most common sources of information was, their veterinarian, internet resources or books, and relatives or friends with no difference in sources of information between groups (Table 3). When deciding on how much to feed the cat, more than half of the owners had never considered it or fed when the cat was asking for it, while a minority fed the cat based on recommendations from the food packaging or veterinarian (Table 3). Treats were given daily or weekly by approximately half of the owners in both groups (Table 3). When treats were given, they were primarily given in addition to the *ad libitum* feeding or daily diet allocation and only a minority of owners reduced the daily food ration or used part of the daily ration for treats (Table 3). There was no difference in frequency of giving treats or relation to the daily diet allocation between the groups.

The majority of owners of both groups had never discussed their cat’s weight with their veterinarian (Table 3) and when it had been discussed, less than 30% of owners of heavy/obese cats had been informed that their cat was overweight. However, the discussion with the veterinarian had more often resulted in a diet change for cats in the heavy/obese group compared with normal weight (*p* < 0.004, Table 3).

### 3.4 Multivariate analysis—cat characteristics and their interaction with indoor/outdoor access

**Figure 1:**
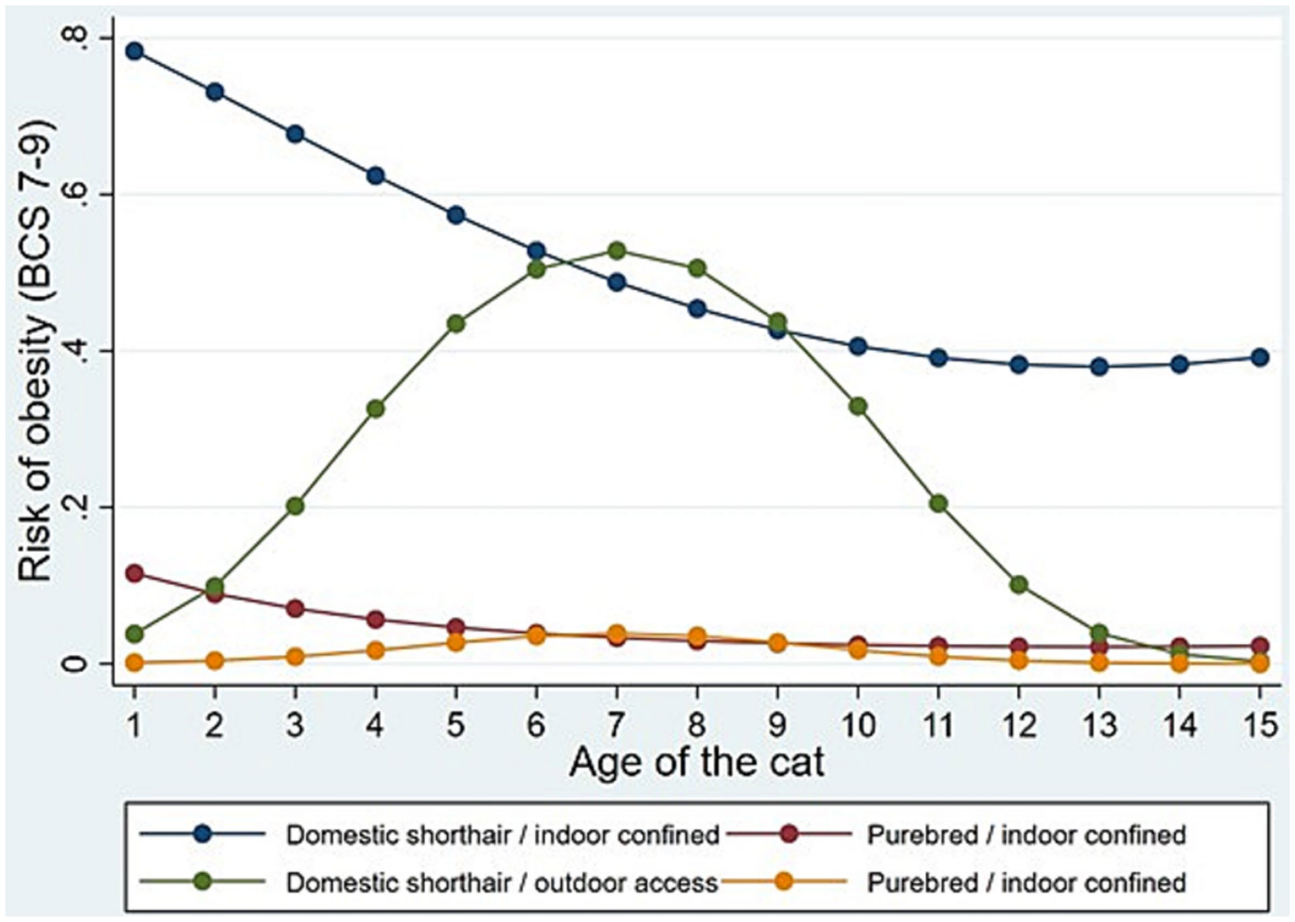
Multivariable logistic regression showing the interaction between the cat’s breed [domestic shorthaired (DSH) or purebred], age and indoor confinement/outdoor access in a cohort of privately owned cats examined in their home environment (*n* = 192).

The multivariable logistic regression returned a statistically significant model [LR chi2 = 88.57 (29); *p* < 0.001; pseudo-R2 = 0.3566; log-likelihood −79.901; *n* = 192]. See omnibus test of model effects in Supplementary Table S2. Three variables relevant to the cat’s environmental and biological characteristics were found to predict the risk of a heavy/obese status (BCS 7–9/9) at the statistically significant level: the cat’s breed (DSH or purebred), age and indoor confinement/outdoor access, respectively. Specifically, there were statistically significant interaction effects between cat age and indoor/outdoor status. In Figure 1, we present how the risk of being heavy/obese is patterned according to the three variables. It is seen that indoor confined DSH cats have a high risk of being heavy/obese (>0.70 predicted probability) already at a very young age (1 year old), and that the risk continues to be high though it steadily decreases until the age of 9–10 years after which the risk plateaus (at around 0.40 predicted probability). The risk for DSH cats with outdoor access is very low (<0.10 predicted probability) at a young age and slowly increases culminating around 7 years of age (at appr. 0.50 predicted probability) after which the risk drops. In contrast, in purebred cats, the risk is in general very low and age very modestly affects the risk of being heavy.

### 3.5 Multivariate analysis—owner characteristics, attachment, perceived cat activity level and feeding practice

The owners, age, education, and weight status did not predict the cat’s risk of being heavy/obese at the statistically significant level. Gender, however, did have a bearing (*p* < 0.01), as the risk was higher in male respondents compared with female respondents. Also, owners’ attachment to the cat was significantly associated with being heavy/obese (*p* < 0.05). Owners of heavy/obese cats were significantly more attached to their cats compared with owners of normal weight cats. Additionally, frequency of meals was identified as significantly associated with the risk of being heavy/obese (*p* < 0.05). Feeding *ad libitum*, meaning that food is always available, was negatively associated with being heavy/obese. Finally, higher cat activity (as reported by the owner) was statistically associated with a lower risk of being heavy/obese (*p* < 0.05). See further details in Table 4.

**Table 4:** Results of multivariate analyses, identifying significant risk factors for cats being heavy/obese [body condition score (BCS) 7–9/9] in 192 privately owned cats evaluated in their home environment.

Variable	Risk of cat being heavy/obese (BCS7-9/9)
Gender of the owner	
Woman	0.32
Man	0.58
Owner-reported activity level of the cat	
Low (1–4)	0.49
Medium (5–7)	0.36
High (8–10)	0.21
Daily feeding	
1–2 times per day	0.42
More than 2 times per day	0.49
Ad libitum	0.28
LAPS	
−1 SD	0.27
Mean	0.35
+1 SD	0.44

Risks are reported in proportions and calculated using Stata’s margins command following the logistic regression.

## 4 Discussion

In this paper, the risk factors for cats being heavy/obese (BCS ≥ 7/9) were investigated based on a sample of cats examined in their home environment by two investigators trained in body condition scoring. Our main findings, complement current knowledge in two ways: firstly we found that owners of heavy/obese cats are on average more attached to their cat and that indoor confinement predispose DSH cats to being heavy/obese from a young age while outdoor access seems to postpone development of overweight.

In light of the existing literature, the discussion will focus on the following risk factors: (1) cat characteristics, with a focus on our findings regarding the effects of age, sex, neutering and breed; (2) owner characteristics and attachment to their cat, with a focus on the effect of the owner’s attachment; (3) feeding management and activity, with a focus on the feeding practices, indoor/outdoor housing and owner perceived cat activity level. Finally, we will conclude by discussing the strengths and limitations of our study.

Regarding cat characteristics, the most important finding of the study concerns the interaction between breed, age and housing where there was a significantly increased risk of being heavy/obese already at 1 year of age for DSH cats if they were indoor confined compared with having outdoor access. DSH cats had a significantly higher risk of being heavy/obese compared with purebreds irrespective of age or housing, but the development in relation to age followed a similar trend for indoor confined purebred cats as for indoor confined DSH cats. Indoor confinement increased the risk of being heavy/obese equally from 1 to 11 years of age, while cats with outdoor access had a gradually increasing risk of being heavy/obese from the age of 3 to 11 years, with a maximum risk around 7 years of age. A study investigating movement patterns of cats with outdoor access found that older cats (8–11 years) walk less than cats aged 4–7 years, this could partly explain the gradual increase in overweight among cats with outdoor access, as their food intake may not be adjusted to a decreasing activity with age (36).

Most other studies have found an increased risk of obesity for DSH or mixed breeds compared with purebred (3, 7, 8, 16, 23) and age has also been reported as a risk factor in several studies, where middle-age (5–12 years) has been most frequently reported (2, 3, 5, 9, 16, 20, 22, 23, 37–41), but also from 1½ year and up (3, 18, 19, 38) and up to 15 years (3) has been reported. One study, evaluating risk factors for having an elevated owner reported BCS at 2 years of age, found being overweight at 1 year of age to be the most significant factor (24). Similarly, a study evaluating *ad libitum* fed colony cats, identified body weight at 1 year of age as the main factor in predicting later overweight, possibly due to a faster growth rate between 3 and 12 month of age compared with the lean colony cats (42). Relating these findings to the current study, it could indicate, that overweight in indoor confined neutered cats is established early in life, and possibly that cats with a less effective appetite regulation may increase both growth rate and fat deposition at an early age if fed unrestricted. Neutering was not identified as a risk factor in the current study, but this is probably due to the very low number of intact cats. Neutering has been identified as a strong risk factor in several previous studies (2, 3, 9, 10, 16, 18–20, 22, 43). Neutering decreases appetite regulation and increase food intake in cats (44, 45) and may further exacerbate the obesity development in indoor confined cats. In the current study, where 70% of the cats were neutered between 3 and 12 months of age, age at neutering was not identified as a risk factor for being heavy/obese, which is in line with a previous study (6). A recent study, however, found a less rapid age-related increase in bodyweight and BCS in cats neutered at the age of 7–12 months compared with less than 4 month and 5–6 months (38). Male sex was not identified as a risk factor in the current study which is in line with some studies (5, 6, 12, 16, 18, 22, 25, 39, 41) but not with other (2, 3, 7, 8, 10, 19, 20, 43). The difference does not seem to be related to study design or countries with a tradition for neutering at an early age. Male cats are larger than female cats, and in the original development of the 9-point BCS scoring system, male cats having the same BCS score tended to have a lower body fat percentage (46). This was however not confirmed in the later validation study in indoor confined cats (47). Because obesity is frequent in both male and female cats, sex should not be a factor in deciding on whether to discuss risks of overweight/obesity with cat owners.

In terms of owner characteristics and attachment to the cat, owners’ attachment to the cat was significantly stronger for the heavy/obese group, even when correcting for the possible confounder of indoor confinement, that may both strengthen attachment and lead to heavier cats. Having a male owner was associated with the cat being heavy/obese, which has not previously been described. A few studies identified having a female owner and owner not working or going to school (28) or owner aged 41–60 years (19) as risk factors. When recruiting owners and cats, we asked that the primary caregiver should respond to the questionnaire to ensure as qualified answers as possible. The number of male owners was 10 in the normal weight and 12 in the heavy/obese group, and it is possible that this finding is not reproducible, but it could reflect gender-related differences in weight perception, feeding management vigilance and use of treats. Owner BMI was not associated with the weight status of the cat. Owner BMI has been associated with obesity in dogs and has been correlated with obese owners’ use of treats in their interaction with their dog (48). Though cat owners frequently give treats, it is likely that giving treats to a cat is less associated with the owners’ lifestyle compared with dogs. In a previous study on cat obesity, BMI of the owner and the owners eating behavior were investigated, and, similar to the current study, there was no association between feline overweight and owner BMI or eating behavior (28). Our finding, that owner attachment was associated with the cat being heavy/overweight, differs from two previous studies (5, 16), one of which used the same LAPS scoring system (16). However, one study found an association with owners feeding treats/table scraps when feeling happy with the cat (24) while another study identified associations with cat acquired to console and encourage owner, owners finding it important to talk with the cat, owner giving in for begging, owner watching the cat eat and finally, using food as treat versus play as treat (28). Further, one study identified that expressing positive and approving attitudes towards feline obesity was positively associated with ownership of obese cats (23).

In terms of feeding and housing management, indoor confinement, was significantly associated with being heavy/obese from a younger age compared with cats having outdoor access. Several studies have found an association between indoor confinement and overweight in cats, including a previous Danish study (6, 16, 18, 21), though others have not been able to confirm this association (2, 8, 9, 12, 19, 22, 41). Furthermore, cats perceived by the owner to have a high activity level were less likely to be heavy/obese. In a study on colony cats, overweight cats were also less active compared with lean cats (49). In a study investigating seasonal effects on energy expenditure in cats either indoor confined or with outdoor access in a temperate climate (New Zealand) during winter and summer, respectively, (50), young cats (3.1 ± 0.59 years) having outdoor access had a higher energy requirement compared with those being indoor confined, both during summer and winter seasons. The cats were fed *ad libitum,* and there was no difference in energy intake between indoor confined cats and cats with outdoor access despite the difference in energy requirement. In the current study, there was a lacking adjustment of feeding practices in relation to cat weight status as the majority of cats were fed *ad libitum*, and it is likely that an excess energy intake in combination with a low activity level may predispose to a faster growth rate and development of overweight at an earlier age in indoor confined cats. Though *ad libitum* feeding was the preferred feeding method for both groups in the current study, less heavy/obese cats were fed *ad libitum*. This could relate to owners of heavy/obese cats having changed feeding regime from *ad libitum* following a veterinary consultation or that owners choose *ad libitum* feeding when there is no perceived weight problem. Feeding *ad libitum* has been identified as a risk factor in two studies (23, 28), while other studies have not found an effect of feeding frequency (2, 5, 8, 9, 19, 23–25, 41). Treats and table scraps were frequently given to the cats and were seldomly calculated as part of the daily diet allocation for both groups. There was no difference between groups with respect to giving treats or not, type of diet, feeding primarily dry diet or how daily diet allocation was determined. A few previous studies have found “feeding treats or table scraps” to be a risk factor (22, 24, 28), while most other studies did not find an association (2, 5, 8, 12, 22, 23, 25, 26, 41, 51–53). Type of food has been identified as a risk factor in some studies, but the identified type of food is inconsistent. Feeding dry food (21, 24), canned food ad libitum (22), feeding primarily premium food (dry or wet) or therapeutic diet (19) and commercial (not therapeutic) dry food has been identified as risk factors, while feeding human grade raw food, freeze dried food and home-made food has been identified as being protective in one study (16). Despite owners being aware of obesity related health risks in general, obesity in one’s own cat was seldom acknowledged and diet regimes seldom changed, with a majority of heavy/obese cats still being fed regular cat food and *ad libitum.* Even when the cat’s weight had been discussed during a veterinary consultation, a resulting diet change seldomly included weight loss diets or programs. This contrasts with the finding that the owners in our study claimed to be willing to change their cat’s diet and increase its activity if it was obese.

The present study has some strengths and limitations that should be considered when evaluating the data. The cross-sectional study design is useful for identifying correlations between different factors and the presence/degree of feline obesity. While the results may provide a basis for future prospective longitudinal studies, great care should, however, be applied before making claims about causality. The studies that previously evaluated risk factors for feline obesity were performed in different populations and used different recruitment strategies, as well as inclusion and exclusion criteria. In some studies, BCS was assessed and reported by clinical staff, but not always by the same individuals, while many studies have relied on the owners assessing BCS. In planning the current study, emphasis was put on limiting bias in BCS assessment. This was done by the BCS scoring being performed jointly by student investigators trained by skilled personnel prior to study start. Furthermore, because the BCS system is poorly validated for use in growing cats, it was decided that only cats above 1 year of age would be included and to avoid including cats whose body condition was a result of factors other than feed management, cats diagnosed with chronic disease or given medication that could affect their appetite were also excluded from the analysis. In the analysis, the cats were divided into two groups based on their BCS, normal weight (BCS4-6/9) and heavy/obese (BCS 7–9/9). The main reason for this division was the indications from recent studies, that BCS 4/9 and 6/9 in cats might not incur the same health consequences as seen with both lower and higher scores (1).

To achieve demographic variation and to account for the fact, that some cats are infrequently visiting their veterinarian and that owners underestimate their cats body condition, cats were recruited through social media and examined in their home environment in both rural and urban areas. A study, focussing on cat welfare in Denmark, was based on a representative sample of the Danish population in 2015 (31). In that study, there was a similar distribution in cat breeds, sex, neuter status, and composition of household, while the current study included more indoor confined cats, multi-cat households, cats in the age range 4–7 years, female owners and owners with lower income and less owners above 65 years of age. These differences indicate that the included cat population may not be completely representative for the Danish cat population. Another possible bias was that in households with more than one cat, the cat closest to 5 years of age were selected. This has selected for a relatively narrow age range, with few geriatric cats and it may have selected for overweight/obese cats in the current cohort. Selecting based on the first letter in the cat’s name would have been a better choice. Furthermore, if we had included all cats in the multi-cat households, we could have allowed for more in-depth analysis for with-in household factors than possible with the current set-up, and this approach could be considered in future studies.

In conclusion, the current study confirmed a high prevalence of being heavy/obese in privately owned cats and it adds to the understanding of risk factors relating to feline obesity, especially highlighting that owners with heavy/obese cats seem more attached to their cats. Furthermore, a significant association between indoor confinement and being heavy/obese already from 1 year of age in DSH cats underscores that preventative measure, including rationing the daily diet allocation and possibly feeding diets with a lower calorie density should be recommended at an early age for all neutered cats, and especially if they do not have outdoor access. Owners wished to support their cats health and also expressed an interest in intervening if their cat was overweight. Unfortunately, restrictive feeding strategies or interventions seemed not to be realised despite veterinary interaction relating to cat weight status.

## Data Availability

The raw data supporting the conclusions of this article will be made available by the authors, without undue reservation.
