# Supplementary material for: Feline obesity is associated with stronger owner attachment, while indoor confinement increases risk of obesity at an early age in domestic shorthaired cats
Source: Front Vet Sci. 2026 Mar 18;13:1757719. doi: 10.3389/fvets.2026.1757719 (PMC13041544; doi:10.3389/fvets.2026.1757719)
Supplement: Supplementary file 1 [file Table_1.DOCX]

**Supplementary material S1 – questionnaire**

*Questionnaire*

New respondent

Cat number:

________________________________________

The first part of the questionnaire concerns specific information about your cat

1. How old is your cat?

_________________ years

2. What sex is your cat?

- Female
- Male

3. Is your cat neutered?

- Yes
- No
- Don´t know

4. When was your cat neutered/castrated?

- 3-6 month of age
- 6-9 months of age
- 9-12 months of age
- Older than 12 months of age
- Don´t know

5. Is your cat purebred or a mixed breed?

- Purebred
- Domestic shorthaired
- Mixed breed
- Don´t know – go to question 6

5a. What breed is your cat? If it is a mixed breed, please note the primary breeds (a maximum of 3)

________________________________________

6. How many cats are there in the household?

- 1 cat
- 2 cats
- More than 2 cats

7. Is your cat being regularly dewormed?

- Yes
- No
- Don´t know

8. How often do you bring your cat to the veterinarian?

- Several times a year
- Once yearly
- Every second year
- Every third year
- Seldom
- Never

9. What does it require for you to bring your cat to the veterinarian (apart from vaccinations or similar)

- That it has severe signs of illness
- That it has minor signs of illness
- Don´t know

The next part of the questionnaire concerns your cat’s feeding routine

10. What do you feed your cat? Please give the approximate ratio of diet provided per day in percentages (for example, 40% home-made and 60% dry food; more than one response is acceptable) The total amount should equal 100%.

- Dry food

_________ %

- Canned food

_________ %

- Home-made

_________ %

- Leftovers

_________ %

- Other (for example BARF - please specify)

_________ %

________________________________________

________________________________________

- Don´t know

If you did not answer dry or canned food, please go to question 13

11. Where do you buy your cat´s food?

- Supermarket
- Pet shop
- Veterinarian, animal hospital
- Online on the internet
- Other
- Don´t know

12. What kind of food do you feed your cat?

- Standard (go to question 7)
- Weight loss diet
- Prescription diet (for example disease or allergy diet, please specify)
- Don´t know

13. How often do you feed your cat per day?

- Once a day
- Twice a day
- More than twice a day
- *Ad libitum*, i.e. the food is accessible at all times
- Don´t know

14. Have you ever sought advice about how to feed your cat?

- Yes
- No
- Don´t know

15. If yes, where did you seek advice about your cat’s feeding? (more than one answer is acceptable)

- Recommendation from veterinarian/veterinary nurse
- Recommendation from pet store
- Recommendation from other people (friends, acquaintances, kennel owners)
- Recommendations from books/magazines/the internet
- Recommendation on the bag of food
- Other ___________________________________________________________
- Don´t know

16. How do you feed your cat?

- The cat is fed depending on it´s appetite, e.g. meowing or begging
- The cat is fed according to recommendations as described above
- The feeding is adjusted based on changes in the cats’s weight
- I have never thought about it
- Other
- Don´t know

17. Does your cat get treats?

- Yes
- No
- Don´t know

17a. How often does your cat get treats?

- Daily
- Weekly
- Seldom

17b. Which of the following statements fits best with the way your cat is given treats?

- Treats are provided in addition to the daily food ration
- Treats are provided in addition to the daily food ration – but the daily food ration is decreased
- The cats’s normal food is used as a treat and is taken from the daily food ration
- None of the above

The next part of the questionnaire concerns your dog’s exercise

18. Which of the following statements fits best?

- The cat is indoor contained
- The cat is kept indoors, but is let out on a leash or under supervision
- The cat is both indoor and outdoor with access to going out
- The cat is only outdoors

18a. How does the cat get outside?

- The cat is being let out/in
- The cat has access to a cat flap or similar

19. Which of the following activities do you do with your cat or does it do by itself?

- Hunting/play
- Climbing tree/scratching tree
- Training (commandoes)
- Other types of activity – please specify _______________________________
- I/we do not train or play with the cat
- Don´t know

20. How would you rate your cat´s activity on a scale from 1 to 10?

- 1 = inactive
- 2
- 3
- 4
- 5 = medium active
- 6
- 7
- 8
- 9
- 10 = very active

The next part of the questionnaire concerns your cat’s body weight and body composition

21. How do you perceive your cat’s body weight?

- Thin
- Underweight
- Normal weight
- Overweight
- Very overweight
- Don´t know

22. Do you know your cat’s body weight?

- Yes
- No
- Don´t know

22a. If yes, what is your cat’s body weight?

_______________ kg

23. Which of the provided pictures (WSAVA body condition scoring chart) best fits with your cats’s body composition?

- Picture 1
- Picture 2
- Picture 3
- Picture 4
- Picture 5
- Don´t know

24. Have you discussed your cats’s body weight with your veterinarian/veterinary nurse?

- Yes
- No
- Don´t know

24b. What did the veterinarian/veterinary nurse rate your cat´s bodyweight as?

- Thin
- Underweight
- Normalweight
- Overweight
- Very overweight
- Don´t know

24b. Has any changes been made following this evaluation?

- Yes
- No
- Don´t know

25. If your cat was evaluated to be overweight during the evaluation today, would you be willing to adjust the cat´s diet?

- Yes)
- No
- Don´t know

26. If your cat was evaluated to be overweight during the evaluation today, would you be willing to change it to a weight loss diet?

- Yes
- No
- Don´t know

27. If your cat was evaluated to be overweight during the evaluation today, would you be willing to change change it´s activity level?

- Yes
- No
- Don´t know

The next part of the questionnaire concerns your knowledge of obesity in cats

18. Do you believe that there are health consequences of obesity in cats?

- Yes
- No
- Don´t know

18a. If yes, what consequences are you aware of?

________________________________________

________________________________________

19. To what extent do you think that a cat being overweight or obese is a problem that requires help from a veterinarian?

- To a very high degree
- To a high degree
- To some degree
- To a low degree
- Not at all
- Don´t know

The following statements relate to your attachment to your cat, it is important that you write to what degree your agree with each statement

33.

a. My cat means more to me than *any* of my friends

Strongly disagree ____ somewhat disagree _____ somewhat agree ____ strongly agree____

b. Quite often I confide in my cat

Strongly disagree ____ somewhat disagree _____ somewhat agree ____ strongly agree____

c. I believe that cats should have the same rights and privileges as family members

Strongly disagree ____ somewhat disagree _____ somewhat agree ____ strongly agree____

d. I believe my cat is my best friend

Strongly disagree ____ somewhat disagree _____ somewhat agree ____ strongly agree____

e. Quite often, my feelings toward people are affected by the way they react to my cat

Strongly disagree ____ somewhat disagree _____ somewhat agree ____ strongly agree____

f. I love my cat because he/she is more loyal to me than most of the people in my life.

Strongly disagree ____ somewhat disagree _____ somewhat agree ____ strongly agree____

g. I enjoy showing other people pictures of my cat.

Strongly disagree ____ somewhat disagree _____ somewhat agree ____ strongly agree____

h. I think my pet is just a cat.*

Strongly disagree ____ somewhat disagree _____ somewhat agree ____ strongly agree____

i. love my cat because it never judges me.

Strongly disagree ____ somewhat disagree _____ somewhat agree ____ strongly agree____

j. My cat knows when I'm feeling bad.

Strongly disagree ____ somewhat disagree _____ somewhat agree ____ strongly agree____

k. often talk to other people about my cat.

Strongly disagree ____ somewhat disagree _____ somewhat agree ____ strongly agree____

l. My cat understands me.

Strongly disagree ____ somewhat disagree _____ somewhat agree ____ strongly agree____

m. I believe that loving my cat helps me stay healthy.

Strongly disagree ____ somewhat disagree _____ somewhat agree____ strongly agree____

n. Cats deserve as much respect as humans do.

Strongly disagree ____ somewhat disagree _____ somewhat agree____ strongly agree____

o. My cat and I have a very close relationship

Strongly disagree ____ somewhat disagree _____ somewhat agree____ strongly agree____

p. I would do almost anything to take care of my cat.

Strongly disagree ____ somewhat disagree _____ somewhat agree ____ strongly agree____

q. I play with my cat quite often.

Strongly disagree ____ somewhat disagree _____ somewhat agree ____ strongly agree____

r. I consider my cat to be a great companion.

Strongly disagree ____ somewhat disagree _____ somewhat agree ____ strongly agree____

s. My cat makes me feel happy.

Strongly disagree ____ somewhat disagree _____ somewhat agree ____ strongly agree____

t. I feel that my cat is a part of my family.

Strongly disagree ____ somewhat disagree _____ somewhat agree ____ strongly agree____

u. I am not very attached to my cat.*

Strongly disagree ____ somewhat disagree _____ somewhat agree ____ strongly agree____

v. Owning a cat adds to my happiness.

Strongly disagree ____ somewhat disagree _____ somewhat agree ____ strongly agree____

w. I consider my cat to be a friend.

Strongly disagree ____ somewhat disagree _____ somewhat agree ____ strongly agree____

*reverse score (for two items)

The next part of the questionnaire concerns you as the owner

22. How old are you?

_________________ years

23. I am:

- Male
- Female

24. What is your postal code?

_________________

25. what kind of housing do you live in?

- Apartment
- Apartment with a garden
- House/townhouse with a garden
- House/townhouse without a garden
- Farm/house in the countryside
- Other
- Don´t know

26. What is your household composition? (Children living at home refers to children who reside there at regular intervals)

- I live with my parents
- I live with my parents together with siblings
- I live alone
- I live with others
- I live alone with my child/children
- I live with my partner without children
- I live with my partner and child/children
- Other

26a. Are there children below the age of 15 years in the household?

- Yes
- No

27. What is the highest level of education you have completed?

- Less than 7 years in school
- 7-8 years in school
- 9-10 years in school
- High school
- Other

28. Have you completed a higher education? (If more than one, please tick the highest educational level)

- No
- AMU/special worker education
- Introductory year for a vocational education
- Completed a vocational education
- Other vocational education (social worker/health education)
- College/Bachelor’s (2-4 years)
- University (more than 4 years)
- Other

29. What is your current main occupation?

- Self employed
- Employee (private or government)
- On leave
- Unemployed (insured)
- Unemployed (not insured)
- Retired
- Working at home
- Studying
- Other

30. What is the current yearly income of your household?

- Up to 100.000 DKK
- 100.000 - 199.999 DKK.
- 200.000 - 299.999 DKK
- 300.000 - 399.999 DKK
- 400.000 - 499.999 DKK
- 500.000 - 599.999 DKK
- 600.000 - 699.999 DKK
- 700.000 - 799.999 DKK
- 800.000 - 899.999 DKK
- More than 900.000 DKK
- I do not wish to answer the question
- Dont´t know

31. What is your height?

___ cm

- Don´t know _______
- I do not wish to answer _______

32. what is your weight?

___ kg

- Don´t know _______
- I do not wish to answer _______
