# Supplementary material for: Feline obesity is associated with stronger owner attachment, while indoor confinement increases risk of obesity at an early age in domestic shorthaired cats
Source: Front Vet Sci. 2026 Mar 18;13:1757719. doi: 10.3389/fvets.2026.1757719 (PMC13041544; doi:10.3389/fvets.2026.1757719)
Supplement: Supplementary file 2 [file Table_2.DOCX]

**Supplemental table S2 (Test of model effects)**

| Source | Type III | | |
| --- | --- | --- | --- |
|  | Wald Chi-Square | df | Sig. |
| (Intercept) | .^a^ |  |  |
| Cat age | 2,994 | 1 | 0,084 |
| cat age * cat age | 3,681 | 1 | 0,055 |
| Cat gender | 1,814 | 1 | 0,178 |
| Breed | 21,159 | 1 | 0,000 |
| Neuter status and age at neutering | 3,623 | 5 | 0,605 |
| Indoor/outdoor | 13,147 | 1 | 0,000 |
| Treats | 3,845 | 3 | 0,279 |
| Daily feeding practices | 6,915 | 2 | 0,032 |
| Number of cats | 3,798 | 1 | 0,051 |
| Cat activity level (Owver-reported) | 7,274 | 2 | 0,026 |
| Interaction Indoor/outdoor*age | 8,316 | 1 | 0,004 |
| Interaction Indoor/outdoor*age*age | 6,177 | 1 | 0,013 |
| LAPS | 5,551 | 1 | 0,018 |
| Owner gender | 7,032 | 1 | 0,008 |
| Owner education | 4,482 | 3 | 0,214 |
| Owner age | 1,222 | 1 | 0,269 |
| Owner weight status | 1,989 | 3 | 0,575 |
